# The use and experience of the national disability insurance scheme for Australians with skeletal dysplasia: a mixed-methods study

**DOI:** 10.1186/s13023-025-03630-6

**Published:** 2025-03-05

**Authors:** Jun Hei Jeremy Lai, Penelope Ireland, Daphne Nguyen, Ashley Woodbury, Verity Pacey

**Affiliations:** 1 Faculty of Medicine, Health and Human Sciences, Macquarie University, Sydney, NSW Australia; 2 Queensland Paediatric Rehabilitation Service, Queensland Children’s Hospital, South Brisbane, QLD Australia

**Keywords:** Skeletal dysplasias, National disability insurance scheme, Experience, Disability funding, Qualitative, Satisfaction

## Abstract

**Background:**

Skeletal dysplasias are rare disorders affecting bone growth and development that impact functional performance. In Australia, the National Disability Insurance Scheme (NDIS) was rolled out in 2016 to support individuals with disabilities access reasonable and necessary supports to promote independence and quality of life. Anecdotally, Australians with skeletal dysplasias report challenges with accessing and using the NDIS but this has not previously been reported in the literature. Therefore, this study aims to explore the use and experience of NDIS for Australians with skeletal dysplasias.

**Methods:**

This is a cross-sectional, mixed-methods study. Eligible participants included adults and children (represented by their parents) with skeletal dysplasias, irrespective of NDIS access. Participants completed an online survey, the Functional Independence Measure (FIM), or WeeFIM for paediatric participants, and semi-structured interviews exploring their NDIS access, use, and experience. Survey responses and FIM/WeeFIM results were analysed using descriptive statistics. Grounded theory approach and inductive thematic analysis was performed on qualitative data.

**Results:**

Of the 14 participants (10 adults, 4 parents), nine (64%) had NDIS access. Six (66.7%) participants with access reported to be satisfied with their NDIS experience, two (22.2%) extremely satisfied, and one (11.1%) neutral. FIM (median 115.5/126, range 104–125) and WeeFIM (median 95.5/126, range 61–124) demonstrated all participants utilised assistance and/or equipment in daily activities. Three key themes identified through interviews: (1) Consistent, process-driven barriers, (2) Inconsistent, person-driven facilitators, and (3) Impact of NDIS.

**Conclusion:**

Despite all participants demonstrating a need for assistance to achieve functional independence, experience and success in accessing the NDIS were varied. Both positive and negative impacts were reported when accessing, or attempting to access the NDIS. To promote more equal and equitable NDIS access for individuals with skeletal dysplasias, NDIS and condition-specific knowledge is recommended for all stakeholders. Finally, further evaluation is needed to ensure future NDIS eligibility changes provide access to those who are potentially eligible but currently rejected.

**Supplementary Information:**

The online version contains supplementary material available at 10.1186/s13023-025-03630-6.

## Introduction

Skeletal dysplasias are a group of over 430 genetic conditions which causes structural abnormality in bone or cartilage, leading to a disturbance in growth of the extremities and/or trunk [1]. Most forms are non-lethal with minimal cognitive involvement. Common non-improving physical impairments can include short stature, disproportionate limbs-to-trunk ratio, macrocephaly, and spinal alignment issues [1–3].These biomechanical impairments can impact upon functional performance, limit activity participation [4, 5] and frequently require lifelong support and management.

In Australia, the roll out of the National Disability Insurance Scheme (NDIS) in 2016^6^ created an opportunity for people with disabilities to access funding that supported maximising independence and quality of life through personal ‘choice and control’ of service selection, and delivery of supports that were considered ‘reasonable and necessary’ by the scheme [6–8]. The NDIS guidelines require applicants to provide evidence that their disability: (i) is caused by an impairment likely to be permanent, (ii) reduces functional capacity, the ability to work/study and social participation; and (iii) is likely to require lifelong support [9]. In efforts to streamline the process for some conditions, the NDIS has a predetermined list of disabilities that are likely to meet the criteria for NDIS approval [10–12] and be accepted more quickly onto the scheme. However, Australians with disabilities that are not listed, including those with skeletal dysplasias, are frequently required to undertake a more comprehensive review process to evaluate their eligibility to receive NDIS support.

It has been suggested that the scale and logistics for identifying, onboarding, and supporting over four million Australians with different disabilities proved more challenging than expected and that the resources required to support this scheme were underestimated [13]. It has also been reported that lack of disability-specific knowledge by NDIS personnel, excessive medical and scheme specific terminology, and inconsistent funding outcomes create additional barriers for individuals when looking to access or use appropriate supports [14, 15]. Individuals with disabilities report feeling stressed and overwhelmed with the challenges of applying, with some people choosing to not apply at all due to the complexities around navigating the scheme [10, 11]. An independent review of the NDIS, undertaken and published ten years after its inception, identified a number of key recommendations focusing on increasing consistency in eligibility criteria based on functional capacity rather than diagnostic groupings [16]. Similar issues have been identified in other countries, where individuals with rare diseases reported similar challenges when seeking eligibility for insurance funding, as well as difficulties accessing appropriate supports due to the lack of clinical knowledge by the stakeholders [17].

Whilst anecdotally, Australians with skeletal dysplasias report challenges with accessing NDIS support and funding [18], this has not been empirically assessed within the literature or evaluated within the population group. Exploring this from a participant perspective may help understand the barriers and enablers associated with attempting to access supports under a centralised scheme. It may also provide key insights into how stakeholders such as potential participants, clinicians, and scheme workers can more effectively advocate for improved experiences and outcomes of disability support for individuals with skeletal dysplasias, and other rare disorders. Therefore, this study aims to understand the use and experience of NDIS for Australians with skeletal dysplasias. It also seeks to understand the factors that influence access approval, usefulness of the supports provided, and overall level of satisfaction.

## Methods

### Research design and ethics

This cross-sectional, mixed-methods study with an explanatory sequential design, involved two parts: (i) Online survey and functional assessment and (ii) Synchronous audiovisual online interviews [19]. Ethics was approved by the Macquarie University Human Research Ethics Committee (Ref: 5202210737833).

### Participants and recruitment

Individuals with a skeletal dysplasia aged 18 years or older, and caregiver/parents of children/adolescents diagnosed with a skeletal dysplasia under 18 years of age were eligible for inclusion. In this study parents/caregivers were not proxy reporters for the children/adolescents diagnosed with a skeletal dysplasia under 18 years of age. Parents/caregivers were providing functional support to their children (measured by the WeeFIM) and were the contact for any interactions with the NDIS (explored within the interviews). Individuals with cognitive impairment, an inability to read, or who lacked sufficient communication in English to provide informed consent or complete the study requirements were excluded from participation. Australian skeletal dysplasias peer advocacy groups provided consent and support to distribute the survey access link via social media and conferences.

### Data collection

#### Survey

The online survey was hosted on Research Electronic Data Capture (REDCap) [20]. Questions gained information on key demographics; NDIS status, NDIS plan reviews, current funded supports, and level of satisfaction of eligible participants’ NDIS experience using 5-point Likert scale. Participants who completed the survey were then contacted to complete the Functional Independence Measure (FIM) or WeeFIM (paediatric version) via phone, Zoom, or in-person interview, by a research member who was trained and accredited by the Australasian Rehabilitation Outcomes Centre (PI, VP or DN). The FIM/WeeFIM are validated tools that assess functional performance on 18 items across self-care, mobility, and cognition, providing a total score out of 126^21,22^. Individuals who are 8 years and older are expected to achieve a score of 126 (indicating complete independence across all items) [21, 22], with age-related normative data available for children under the age of 8 years [23].

#### Interview

Following completion of the survey and functional assessment, participants completed an interview conducted by two team members (JL and/or VP). This study followed the Consolidated Criteria for Reporting Qualitative Research (COREQ) checklist for guidance [24]. A semi-structured interview script was designed to explore participants’ knowledge regarding NDIS, experiences during their application process, the most valuable and most wanted supports for their skeletal dysplasia, and what their overall best and worst experiences related to accessing and using NDIS have been. For those participants with NDIS access, additional questions were included to explore the utilisation of funding and plan review processes. Interviews were conducted and recorded on Zoom, with transcription. Transcripts were verified by a research team member and returned to participants for approval.

### Data analysis

Quantitative statistical analysis of survey and FIM/WeeFIM data was performed using Microsoft Excel. Standard descriptive statistics (frequency, median, and range) were used due to the small sample size. Geographical location was categorised using the Modified Monash Model 2019, to showcase if participants lived in a metropolitan, regional, or rural area [25]. Thematical analysis of the qualitative data was performed using the grounded theory approach, and four team members (JL, VP, AW, PI) independently performed a constant comparative analysis for coding and category development [26]. All research team members are practising physiotherapists with experience working in paediatrics (VP, PI 20 + years, JL, AW 2–3 years) and with NDIS participants. JL, VP and AW have experience with qualitative research, and VP and PI are experienced clinicians and researchers working with individuals with skeletal dysplasias over a number of years.

Themes and subthemes were developed through inductive theoretical sampling, and agreed upon until no new themes were emerging from the analysis. Furthermore, participants were recruited until no new and relevant knowledge was obtained, indicating that data saturation was reached [24, 26].

## Results

### Quantitative results: Participants

Fourteen participants (10 females) completed the study. The median adult age was 37.5 years (range 20–68 years), with a median height of 139 cm (range 105–155 cm). Of the four children (2 females), the median age was 8.5 years (6–12 years), and the median height was 105 cm (range 93–110 cm) (Table 1). No parent or caregiver reported having a form of skeletal dysplasia.

**Table 1:** Participant demographic data

All Participants	*n* = 14 (%)
Adult	10 (71.4)
Child	4 (28.6)
**Diagnosis**	
Achondroplasia	6 (42.9)
Osteogenesis Imperfecta	2 (14.2)
Pseudoachondroplasia Multiple Epiphyseal Dysplasia Cartilage Hair Hypoplasia Jeune Syndrome 3 M Syndrome Primordial Dwarfism	1 (7.15) 1 (7.15) 1 (7.15) 1 (7.15) 1 (7.15) 1 (7.15)
**Location**	
Metropolitan	10 (71.4)
Regional	2 (14.2)
Large rural town	1 (7.2)
Medium rural town	1 (7.2)
**Participants with NDIS access**	***n*** ** = 9 (%)**
Adult	5 (50)
Child	4 (100)
**Number of years with NDIS access**	
1–2	2 (22.2)
2–5	6 (66.7)
5+	1 (11.1)
**NDIS satisfaction**	
Neutral	1 (11.1)
Satisfied	6 (66.7)
Extremely satisfied	2 (22.2)
Supports provided by NDIS*	
Equipment	8 (88.9)
Therapy	7 (77.8)
Home modifications	7 (77.8)
Car modifications	6 (66.7)
Occupational supports	6 (66.7)
Care/ Respite	5 (55.6)
Other Supports**	4 (44.4)

*As participants could select more than one support, total percentages exceeded 100%. ** Other supports included: Clothing alterations, hydrotherapy, social support, and community participation

**Fig. 1:**
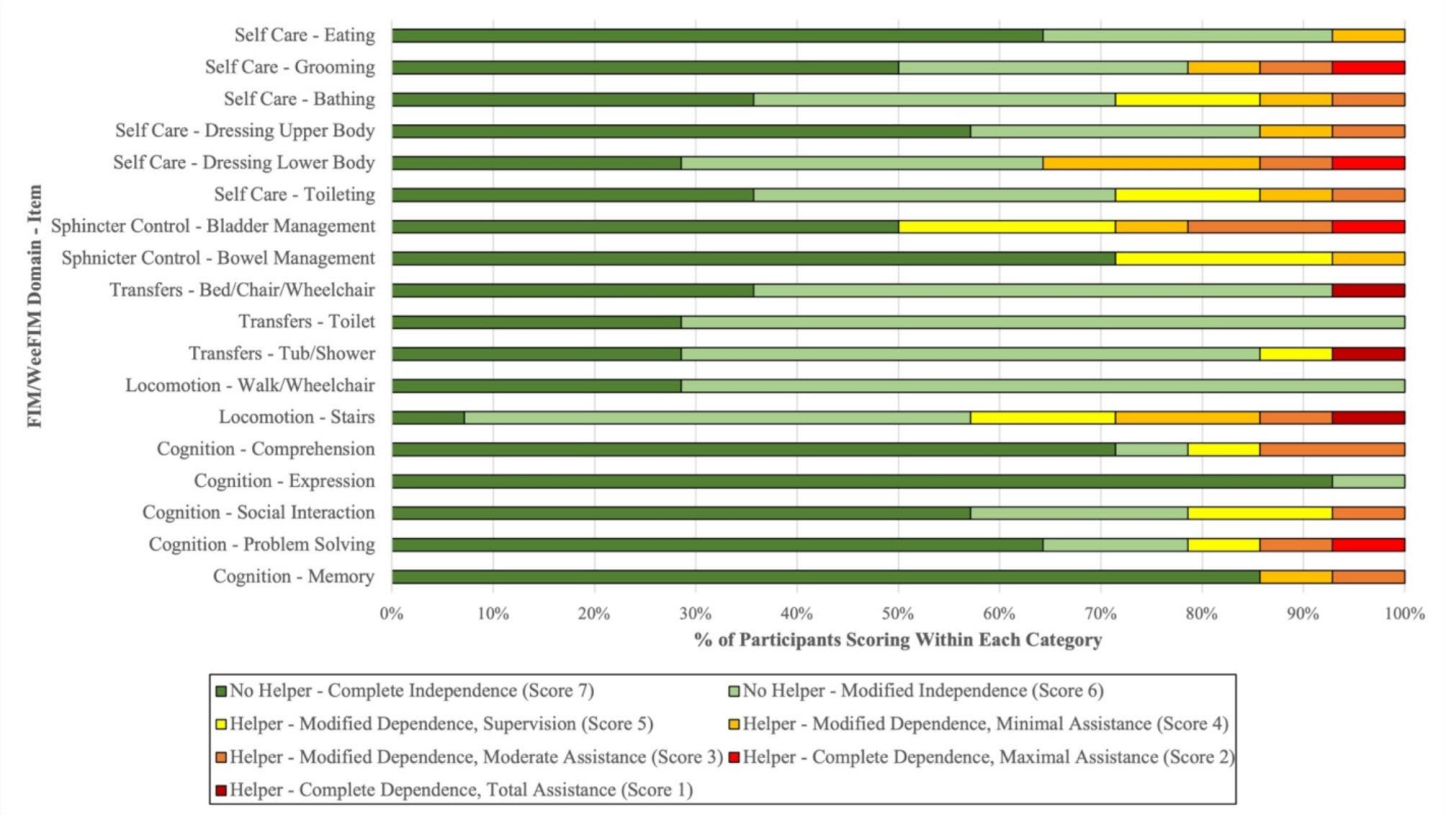
FIM and WeeFIM Scores for Participants with Skeletal Dysplasias

The median FIM score for adults was 115.5 (range 104–125). The median WeeFIM score for children was 95.5 (range 61–124). No domain and corresponding items demonstrated complete independence scores (score 7) from all participants (Fig. 1). Participants scored lowest in mobility related domains, with 29% (*n* = 4) achieving complete independence in Transfers– Toilet, Transfers– Tub/Shower, and Locomotion– Walk/Wheelchair, and 7% (*n* = 1) achieving complete independence in Locomotion– Stairs. Less than half the of participants achieved complete independence for Bathing (*n* = 5), Dressing Lower Body (*n* = 4), and Toileting (*n* = 5) within the Self-Care domain. Over half of the participants achieved independence within each item of the Cognition domain.

Figure 1. FIM/WeeFIM scores that participants achieved on each domain-item (*n* = 14).

### Qualitative results: Overarching themes

Three overarching themes were derived from the interview data suggesting that engagement with the scheme was associated with; (i) Consistent, process driven barriers, (ii) inconsistent, person driven facilitators and (iii) positive and negative impact of NDIS. Six subthemes (two within each theme) were then categorised to capture nuanced concepts within each theme (Table 2).

**Table 2:** Representative participant quotes for themes and subthemes

**1. Consistent, Process Driven Barriers**
*1.1 Challenges in navigating the NDIS*
“I didn’t understand it. I had no idea what was what it was all about. That was fairly confusing. And that’s when I did get the plan… the departments and the sections where everything went and how you claimed what and…what went into what area, and what the funding was exactly for, was just a bit confusing in the beginning.” *(Adult accessing the NDIS for 2–5 years*,* living in metropolitan area)* “…what I’ve heard from parents or from work, unfortunately, it’s, you’ve got to put the right keywords in. Into your application. And if you don’t do that, you won’t get what you need…” *(Adult not accessing the NDIS*,* living in metropolitan area)* “I just think the worst part is waiting, waiting for… the house household supports… to be approved, waiting for the process… The OT’s got to write a report and… send it to [the NDIS], and then that’s going to be approved and blah blah blah… Honestly… I think we first started this process of the bathroom… in the second year and we’re now, you know, entering the fourth year soon. So it is too long…” *(Adult accessing the NDIS for 2–5 years*,* living in large rural town)* “… It is down to the individual that you get with the NDIS as to how you go. 100% driven by that person… The first year we did it, I think we dealt with three people because they kept changing… You know, so there’s no consistency.” *(Parent of child accessing the NDIS for 2–5 years*,* living in metropolitan area)* “Because I’ve heard that a lot that has been families out there and it’s been, in particular, adults with a skeletal condition, really struggle with [applying for NDIS] and I think that that’s unfair because how can some kids just get, breeze through it, and be accepted with no issues, and some can’t? Like why I don’t understand how you pick and choose.*” (Parent of child accessing the NDIS for 2–5 years*,* living in metropolitan area)*
*1.2 Poor knowledge of skeletal dysplasias*
“… Obviously it’s like a rare condition, it’s not something that every second person has. And I suppose like I go to [health professionals] for advice, and questions and if they don’t know information, and they don’t have knowledge of [their] condition. Well then, they can’t help me. Therefore, X doesn’t get help.” *(Parent of child accessing the NDIS for 2–5 years*,* living in metropolitan area)* “… I think people are really unaware of what we need, and that the challenges that present… I think maybe they think that, you know, oh short statured people are just smaller but they can do x, y and z like everybody else can.” *(Adult not accessing the NDIS*,* living in metropolitan area)* “X has got achondroplasia. And apparently [the NDIS] don’t recognize that as a disability. Well I’m like, okay, that’s a physical disability, and it’s a permanent disability, and it’s a non-improving disability so how it’s not accepted or recognized…” (*Parent of child accessing the NDIS for 2–5 years*,* living in metropolitan area)* “I think it was, thought it was a little bit challenging, because [the NDIS] didn’t really know much about [their] condition… So having to provide a lot more in-depth details of [their] condition, I think was a little bit difficult and challenging…” (*Parent of child accessing the NDIS for 2–5 years*,* living in metropolitan area)*
**2. Inconsistent**,** Person Driven Facilitators**
*2.1 Person centered approach*
“He [NDIS personnel] actually had taken the time to read through everything. And he already knew a lot about X before I went in, which I felt was good because again X wasn’t just a number. He was treating X as an individual person, and he generally wanted to help.” *(Parent of child accessing the NDIS for 2–5 years*,* living in metropolitan area)* “… I’ve had quite a few nice [NDIS personnel] over the phone. that did seem genuinely, they wanted to help with whatever I was, was needing.” *(Adult not accessing the NDIS*,* living in regional area)* “… The lady that did [the plan review] this year she was outstanding. She knew her stuff, she knew how to point you in the right direction without leading you there and telling you that what you needed to do. She would ask questions as she went through to say, well, ‘does X manage with this? So how does X find this’…” *(Parent of child accessing the NDIS for 2–5 years*,* living in metropolitan area)* I’ve luckily found people who are willing to learn and willing to listen… the last thing you want is you know, someone doing the wrong thing by your kid and then you end up with injuries or now the therapy isn’t actually doing anything.” *(Parent of child accessing the NDIS for 5 + years*,* living in metropolitan area)* “… just disappointed and frustrated by the system… the rule based approach… not really having a qualitative impression of peoples’ needs…” *(Adult accessing the NDIS for 1–2 years*,* living in metropolitan area)*
*2.2 Knowledge is necessary*
“I wanted more information but [the NDIS personnel] didn’t tell me exactly what so it kind of felt like the people on the phone didn’t really understand what the process was themselves, either.” *(Adult not accessing the NDIS*,* living in regional area)* “And yeah, and also because I’m slightly more experienced in [plan reviews]. It was easier for me to explain what I needed and what I didn’t need… I’ll definitely be more aware if there are other issues that arise, and be able to deal with it properly rather than just hoping for the best…. Probably the word to use is over the last three years, I’ve gained more experience and knowledge of the NDIS.” *(Adult accessing the NDIS for 1–2 years*,* living in medium rural town)* “… So I guess the more knowledgeable your therapist is, the better, especially with NDIS. Makes your whole experience a lot better than ones that don’t know anything and you end up having to do the work.” *(Parent of child accessing the NDIS for 2–5 years*,* living in metropolitan area)* “What most people think straightaway is that you cannot reach things…but that’s the limit to their understanding… The more I am involved in [the NDIS] process, the more I’m able to articulate exactly the problems we have on a daily basis.” *(Adult accessing the NDIS for 2–5 years*,* living in large rural town)* “[Health professionals having knowledge of skeletal dysplasia is] quite important for me… because… you’re dealing with something that’s just a little bit different to what you’re normally dealing with… We don’t see the world like X does, through X eyes.” *(Parent of child accessing the NDIS for 2–5 years*,* living in metropolitan area)*
**3. Impact of NDIS**
*3.1 Benefits of NDIS access approval*
“The best part for me is getting the support that I need. So I can be independent.” *(Adult accessing the NDIS for 2–5 years*,* living in regional area)* “… Knowing that X’s got that support available… throughout the whole year, which is nice and rebooked, and it’s locked in and we’re not on waiting lists… You know that it’s covered and that the financial support’s there to to keep X running through the whole year… when X needs it. So that definitely… is reassuring.” *(Parent of child accessing the NDIS for 2–5 years*,* living in metropolitan area)* “… the fact that I know as I get older… I’m probably going to need more help, and knowing that [NDIS access] will be available and… if I need to have an urgent review… I know that it can be done and will be done… it’ll help me for whatever my needs in the future will be.” *(Adult accessing the NDIS for 1–2 years*,* living in medium rural town)* “I don’t feel that X would be where X is today living [their] life… as independent that [they’ve] ever been. If we didn’t have access to those therapists, X definitely wouldn’t be where X is.” *(Parent of child accessing the NDIS for 2–5 years*,* living in metropolitan area)* *“*… maintaining the house, shopping and cooking… driving me around places which interrupts the day… [my partner] still, you know does all my personal care and helps me dress and so on. But if there’s an issue like [my partners’] not well or… hasn’t got the time… I can access um the NDIS to provide somebody to help me…with my personal care or help me dress…” *(Adult accessing the NDIS for 1–2 years*,* living in large rural town)*
*3.2 The burden of the NDIS system*
“My close girlfriend, um, has a daughter… older than X, and she applied for NDIS… to get assistance with speech and OT in preparation for schooling… And she was rejected… So I think I was a little bit disheartened by that because we were able to get access and it really benefited X, whereas she wasn’t able to and she had to go the longer alternative way to seek other help…” *(Parent of child accessing the NDIS for 2–5 years*,* living in metropolitan area)* “I’m trying to learn about X’s condition as it is, and then trying to get assistance and it’s very overwhelming… And then with the worry that if it was rejected, how was I going to continue to get the needs that X required?” *(Parent of child accessing the NDIS for 2–5 years*,* living in metropolitan area)* “As a parent you of course always focus on what your child can do… This is very different… you are literally looking at the glass, half empty the whole way through the form, which is actually quite depressing to be honest and not the way you normally think…” (*Parent of child accessing the NDIS for 2–5 years*,* living in metropolitan area)* “So it’s been very hard trying to advocate… And you’ve got other people coming to you, asking for help because they see what you’re doing. And you’re saying, hang on, I’m just a, you know, I’m just a patient…I’m only able to share with you what I experienced…” *(Adult not accessing the NDIS*,* living in metropolitan area)*

### Theme 1: Consistent, process-driven barriers

Participants, irrespective of their status of NDIS access approval, consistently reflected process-driven barriers with respect to access, use, and experience of NDIS funding.

#### Subtheme 1.1: Challenges in navigating the NDIS

All stakeholders reported a lack of knowledge and awareness of how to successfully navigate the NDIS. Challenges were reported in understanding the application and onboarding process, understanding what supports are available and accessible, and how scheme specific terminology plays a vital role in success. Participants identified inconsistencies in scheme acceptance and funding support levels between NDIS applications (despite applying with the same diagnosis), which acted as a barrier towards achieving positive outcomes. Those participants who were accepted and approved for funding found it difficult to access supports such as therapy or home modifications within the plan duration due to limited availability of services and allied health professionals. Unused funds were reported to be at risk of being reduced in future plan reviews. Across all stages of the NDIS (application, plan development and plan utilization), participants identified that scheme specific ‘jargon’ and terminology influenced the use and experience for all participants and was a consistently reported barrier.

#### Subtheme 1.2: Poor knowledge of skeletal dysplasias

Data from the interviews identified a consistent lack of knowledge and awareness about skeletal dysplasias across all stakeholders. Since skeletal dysplasias are not currently included in the predetermined lists of disabilities that may gain streamlined access, approval decisions rely on NDIS personnel who frequently have inconsistent or poor understanding of potential functional difficulties that individuals with rare disorders may have. This lack of knowledge may then impact upon access and/or funding decisions. Another barrier towards positive NDIS outcomes were reported to be the participants’ (or parents who represent their children) limited knowledge in explaining the challenges related to their (or their childs’) skeletal dysplasias when attempting to justify the reasonable and necessary case for funding/equipment to NDIS personnel. Furthermore, Finally, as healthcare professionals are required to provide justifying evidence for NDIS applications and ongoing plans, those with limited knowledge and awareness of skeletal dysplasias were also seen as a barrier due to insufficient or inappropriate advocacy for resources.

### Theme 2: Inconsistent, person-driven facilitators

Participants who encountered or possessed certain person-driven factors that facilitated an improved experience of accessing NDIS. However, it was the variable access to facilitators that influenced a participants’ use of the NDIS.

#### Subtheme 2.1: Person-centered approach

Responses from participants highlighted the importance of a person-centered approach with respect to stakeholder interactions throughout the NDIS experience. Active efforts to advocate, problem solve, empathize, and demonstrate genuineness were seen as desirable characteristics and was potentially sufficient to outweigh limited knowledge in creating a positive impact and experience. However, opportunity to interact with a person-centric stakeholder and create a positive experience was reported to be inconsistent across the entire NDIS process.

#### Subtheme 2.2: Knowledge is necessary

The notion that knowledge of both the NDIS processes and skeletal dysplasias is necessary to a successful onboarding to the scheme emerged from multiple perspectives. Stakeholders equipped with the knowledge and experience of how to successfully navigate through the NDIS system were reported to facilitate positive outcomes. Similar results were identified for those stakeholders who were knowledgeable of skeletal dysplasias and its implications towards requiring NDIS supports. Interactions with individuals who are unwilling or unable to understand a potential participant’s condition generally resulted in poorer outcomes. Even so, the participants’ possession of NDIS and disability knowledge, and/or the interaction with a knowledgeable stakeholder was found to be inconsistent, resulting in both varied NDIS use and experience.

### Theme 3: Impact of NDIS

All participants expressed both positive and negative impacts when using and accessing the NDIS.

#### Subtheme 3.1: Benefits of NDIS access approval

Participants reported that the benefits of NDIS approval potentially contributed to improved independence and quality of life. Successful NDIS participants noted a sense of reassurance that they (or their child) would have ongoing financial support and access to allied health input. Participants identified that home modifications, therapies, cleaning and transport services, support worker accompaniment, assistive technologies like step stools, walking aids, and extended-reacher tools as useful supports provided by the NDIS.

#### Subtheme 3.2: The burden of the NDIS system

Conversely, interview data suggested an ongoing sense of burden for participants across all stages of the NDIS process. Participants perceived that NDIS access was a ‘game of chance’, which further created mixed feelings of guilt, luck and gratitude when participants gained approval whilst their peers may not. Despite successful efforts to be enrolled in the scheme, participants reported facing the same difficulties and challenges upon every plan review process, where they were required to re-advocate for themselves with a disability-focused, non-improving outlook to maximize funding and continue to receive supports. In addition to the sense of responsibility, this emotional burden was particularly emphasized by parents, as the strong focus on disability and impaired performance is contradictory to the way they generally perceived their child.

## Discussion

This study found that the use and experience of NDIS for Australians with skeletal dysplasias is dependent on a complex interplay between consistent process-driven barriers and inconsistent person-driven facilitators, which can both positively and negatively impact participants. This is the first study that describes the NDIS experience for this population group, with results mirroring other qualitative research conducted from the perspectives of parents, carers, NDIS service providers and participants with chronic disabilities [8, 27–30]. Findings suggest that consistent issues like difficulties in understanding and navigating a complex NDIS process, poor stakeholder knowledge of individual disabilities, lack of consistency in creating a person-centered approach, and consequent emotional and financial impacts, are likely to be reflected amongst NDIS users irrespective of disability or geographical location.

The NDIS was designed to maximise the independence and participation of individuals with disabilities within the Australian society [31]. All participants in this study demonstrated the need for assistive equipment and/or additional assistance, and require more time compared to non-affected peers when completing activities of daily living when evaluated using standardised measures. Despite the recognized and recorded daily impact related to having a form of skeletal dysplasia, NDIS approval was noted to be varied for participants. One factor that may have influenced NDIS eligibility may have been due to the age of participants at time of application. Scheme acceptance and funding was reported by the four children in this study, while only half the adult participants were successful in gaining NDIS access. Children under the age of six with evidence of developmental delay without a diagnosis, or under the age of seven with evidence of a specific disability as noted in the identified listings (the only form of skeletal dysplasias included is Osteogenesis Imperfecta) may have access to NDIS services under the early intervention pathway [10, 32]. In contrast, applicants beyond seven cannot access the early intervention pathway despite having the same diagnosis and must be evaluated using different eligibility criteria. Given the inconsistency of knowledge about rare and complex disabilities amongst NDIS staff and healthcare professionals, adult applicants may receive varied approval decisions dependent on specific individuals they interact with. This can potentially result in an inequitable and inconsistent application experience when compared with the early intervention pathway available to child applicants with the same diagnosis [33].

The online survey data within this study elicited positive NDIS satisfaction ratings from all participants who were accessing the scheme. However, during qualitative interviews, participants with NDIS access highlighted that while financial support was appreciated, the supports provided remained inappropriate or insufficient to promote independence, reach goals, and achieve greater quality of life. While knowledgeable and person-centric advocates (participants, parents, NDIS stakeholders) were reported to facilitate improved NDIS access outcomes and experiences, individuals with these traits were reported to be inconsistent across the system. This finding is similarly reported from the perspectives of parents of children with more prevalent disabilities, as well as allied health workers in rural areas of Australia, suggesting that this problem exists irrespective of location or disability, and affects more than just the participants themselves [28, 33]. Hence, it may be even more disadvantageous for Australians with rare disorders to access the appropriate supports as they must negotiate and self-advocate at every stage of their journey due to a lack of condition and disability specific knowledge of key stakeholders, as echoed by other rare disease participants/representatives both in and out of Australia [17, 34].

It is widely recognized that the NDIS benefited many Australians with disability by providing access to equipment, home modifications, therapy and in home supports. This was echoed in the 2021–2022 NDIS annual report where an overall positive experience was reported by current NDIS users, although this group was largely represented by participants diagnosed with more common diagnoses such as cerebral palsy or spinal cord injury, or those fulfilling requirements for streamlined access [31]. In contrast, this study found a more nuanced NDIS experience for a population of individuals with rare disorders that are not prevalent and do not meet the streamlined or automatic acceptance criteria set out by the agency. Despite efforts to appraise the effectiveness of the scheme, the expressed positive sentiment may not have accurately reflected the totality of the NDIS experience for a broader range of participants with rare and poorly understood disorders (including those who had access denied), who appear to face greater challenges when accessing and using the scheme. Furthermore, the current published qualitative literature about the impact of NDIS mostly represents families/parents of children with physical disability, service providers/participants in rural areas, single-gendered representation, or participants with psychosocial disabilities [27, 28, 30, 33, 35, 36]. In contrast, this is the first qualitative study to involve adults and children of both genders, diagnosed with the same rare disorder, who live in metropolitan, regional, and rural areas. While there is only one other publication that has examined individuals with a rare disorder and included self-reporting adult participants [34], it did not showcase the depth of nuance and complexities of NDIS use and experience that this study found.

A recent independent review of the NDIS supports findings from this study by identifying a lack of transparency and equity in the application process and acceptance for participants onto the scheme [16]. This review recommended improvements in fairness and consistency across participant pathways through evaluation of functional capacity rather than medical diagnosis, removal of access lists of disabilities that granted automatic/streamlined eligibility, and a shift towards simpler and clearer application processes. If the review recommendations and actions were implemented, all participants in this current study would have successfully gained NDIS access, as they all demonstrated a reduction in functional capacity using standardised WeeFIM/FIM assessment.

With multiple legislative and policy changes currently in the process of being implemented, future evaluation is recommended to ensure these changes achieve simpler, streamlined and more equitable access for eligible participants, including those with rare conditions. Beyond the recommendations and actions, research has also shown that NDIS staff lack formalised disability-specific training [37]. It may be beneficial to develop a training package that assists NDIS staff to better understand rare diseases and their implications and how to provide a consistent patient centric experience for this population. It may also be beneficial for the National Disability Insurance Agency to utilise rigorous qualitative approaches to capture a greater understanding of participant satisfaction of the NDIS and separate the satisfaction ratings between participants with more common disabilities and rare disorders to ensure accurate representation. Furthermore, ongoing review and research to ensure that eligibility changes to access the NDIS are achieving the desired outcomes of improving access processes, should also explore the experiences of potentially eligible applicants who are initially attempting to access the scheme, or have been rejected.

A strength of this study is its mixed-methods approach, allowing it to explore the depth and breadth of nuanced concepts that survey responses alone could not achieve. This study also had limitations. A recruitment bias may have existed as many participants were recruited via peer advocacy groups, who may have different perspectives on disability and increased need for NDIS funding compared to non-advocacy group members. Additionally, two research members have longstanding professional relationships with individuals with skeletal dysplasias and relevant peer advocacy groups, potentially leading to a bias in achieving results advocating for better disability support. To reduce this potential bias, two research team members not associated with this patient population or advocacy groups, also performed data analysis.

Findings from this study may support improvements to those insurance or disability services outside Australia providing supports to individuals with low incidence and rare forms of disability such as skeletal dysplasias. The provision of simplified and consistent pathways to scheme access, clear knowledge and understanding about processes and rare forms of disability, and stakeholders trained in provision of a patient-centred approach would support individuals to have a more positive experience and reduce the burden of care associated with repetitive and complex system processes.

## Conclusion

The use and experience of NDIS funding for Australians with skeletal dysplasias is deeply complex, and exhibits consistent inconsistencies at every stage of the process as well as the outcome. As a first step towards improvement, there is a need to increase the knowledge and awareness of both the NDIS and skeletal dysplasias for all stakeholders. This may promote more positive and valuable experiences involving the NDIS, such that individuals with rare disorders like skeletal dysplasias can gain equitable and consistent support from a scheme that aims to provide this, while reducing the burden upon advocates and participants. Finally, further evaluation is needed to ensure future NDIS eligibility changes provide access to those who are potentially eligible but currently rejected.

## Electronic supplementary material

Below is the link to the electronic supplementary material.

Supplementary Material 1

## Data Availability

All data collected and/or analysed during the current study are available from the corresponding author on reasonable request.

## Abbreviations

NDIS: National Disability Insurance Scheme
FIM: Functional Independence Measure
WeeFIM: Functional independence measure for children
REDCap: Research Electronic Data Capture
COREQ: Consolidated criteria for reporting qualitative research
