# Supplementary material for: The use and experience of the national disability insurance scheme for Australians with skeletal dysplasia: a mixed-methods study
Source: Orphanet J Rare Dis. 2025 Mar 5;20:105. doi: 10.1186/s13023-025-03630-6 (PMC11883912; doi:10.1186/s13023-025-03630-6)
Supplement: Supplementary file 1 — Supplementary Material 1 [file 13023_2025_3630_MOESM1_ESM.docx]

**Supplementary Material 1: Thematic Map**


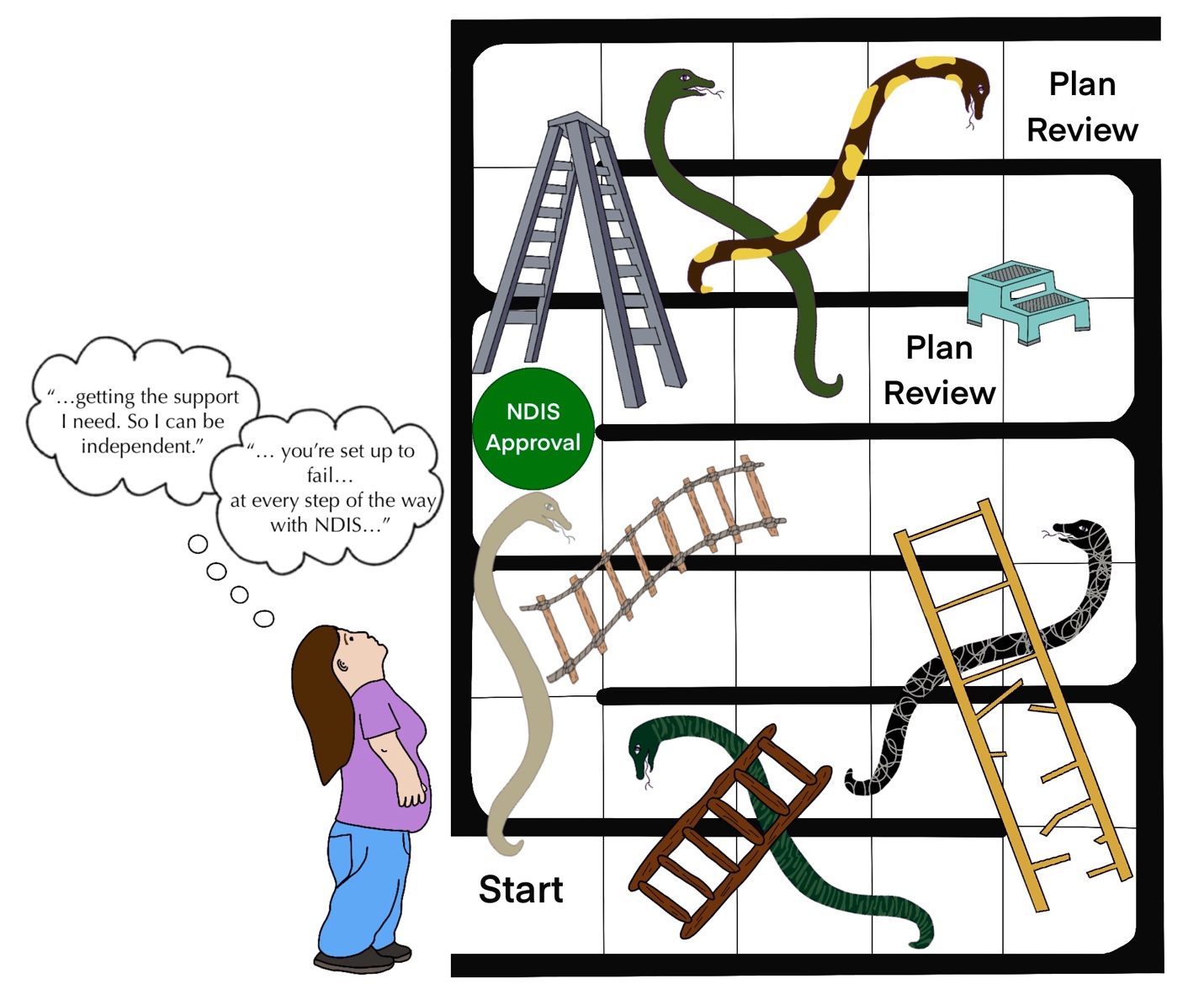


Supplementary Material 1. A thematic map of the possible interactions between themes based on the results. Theme 1 is represented by the snakes. Although they are uniquely designed to exhibit different subthemes, they are all uniform in length, emphasising the consistency of barriers. Theme 2 is represented by the ladders. Similarly, the distinct designs represent different subthemes, but the inconsistency in facilitation is expressed by the varying lengths and characteristics of each ladder. Theme 3 is represented by the thought bubbles, where participant quotes are presented to showcase the positive and negative subthemes. The open-ended board game signifies that NDIS involvement and experience has no end, but remains as a continuous journey.
